# Development and Validation of a Nomogram based on cell growth-related Biomarkers for Oral Squamous Cell Carcinoma

**DOI:** 10.7150/jca.54475

**Published:** 2021-06-22

**Authors:** Yanjie Shuai, Yuansheng Duan, Mengqian Zhou, Kai Yue, Dandan Liu, Yan Fang, Yuxuan Wang, Yansheng Wu, Ze Zhang, Xudong Wang

**Affiliations:** Department of Maxillofacial & E.N.T oncology, Tianjin Medical University Cancer Institute & Hospital, Key Laboratory of Cancer Prevention and Therapy, Tianjin Cancer Institute, National Clinical Research Center of Cancer, Tianjin, China.

**Keywords:** oral squamous cell carcinoma, immunohistochemistry, biomarkers, nomogram, prognosis

## Abstract

**Purpose:** We aimed to develop a prognostic nomogram based on immunohistochemistry (IHC) biomarkers of patients with oral squamous cell carcinoma (OSCC).

**Methods:** A total of 294 patients were enrolled in the study. The least absolute shrinkage and selection operator (LASSO) Cox regression model was performed to develop a combined IHC score (IHCs) classifier.

**Results:** Five biomarkers, specifically c-Met, Vimentin, HIF-2α, VEGF-c, and Bcl-2 were extracted. Then, an IHCs classifier was developed, and patients were stratified into high- and low-IHCs groups. In the training cohort, the 5-year overall survival (OS) was 62.1% in low-IHCs group and 28.2% in high-IHCs group (*P*<0.001). The 5-year OS was 68.6% for the low-IHCs group and 28.4% for the high-IHCs group in the validation cohort (*P*<0.001). The area under the ROC curve (AUROC) of the combination of the IHCs classifier and TNM stage was 0.746 (95% CI: 0.658-0.833) in the training cohort and 0.735 (95% CI: 0.651-0.818) in the validation cohort, respectively.

**Conclusions:** The nomogram could effectively predict the prognosis for patients with OSCC and may be employed as a potential tool to guide the individual decision-making process.

## Introduction

Oral cancer is a common malignancy in head and neck cancer. It is the eighth most common cancer in males, and the incidence rate increases annually 1. Oral squamous cell carcinoma (OSCC) is the predominant histological type, accounting for approximately more than 90% of oral cancers. The histological differentiation of OSCC could be different from that of well-differentiated keratinized to undifferentiated nonkeratinizing carcinoma, which would have a significant difference in prognosis 2, 3. Although improvements in the treatment have evolved from a surgical-based treatment model to multimodal therapies, the prognosis of patients with OSCC remains poor, with the 5-year overall survival (OS) rate ranging from 15% to 60% 4, 5.

The current American Joint Committee on Cancer (AJCC) TNM staging system and important prognostic indicators are widely used as guidelines in the decision-making process 6. The depth of invasion (DOI) was added to the surface dimensions and local extent of the tumour as the required parameters for primary tumour staging in the eighth edition of the AJCC staging system, making the TNM staging more accurate for the assessment of the tumour. However, in our clinical practice, it was usually found that the survival outcomes of patients with the same TNM staging could differ greatly even if they received similar treatments, indicating that the current TNM staging system could not provide individualized prognostic information for patients, and this might be related to the tumour heterogeneity of OSCC. Therefore, a supplement to TNM staging system may assist in evaluating the tumour growth, invasiveness, and sensitivity to treatment for better predicting the prognosis of patients with OSCC.

With the recognition of molecular biology, many genes/proteins that play an important role in the process of carcinogenesis have been discovered. A molecular biomarker could assist in the diagnosis of disease, help in the treatment decision making process, provide information about the tumour's sensitivity to the treatment and indicate the possible prognostic effects based on response to therapy. Recently, many studies of the OSCC biomarkers have been published; however, OSCC biomarkers are still in the early stage of development, and most of them have been investigated separately, and are far from being combined with clinical applications.

Therefore, a classifier, which combines the expression of molecular biomarkers with the TNM staging system, is urgently needed to classify patients with different risks of relapse and metastasis. This study included several biomarkers that have been reported to be involved in biological processes during carcinogenesis, such as epithelial-mesenchymal transition (EMT), extracellular matrix disassembly, angiogenesis, and cellular regulatory framework involving activation of crucial signalling pathways and enduring hypoxia. Then, we aimed to develop and validate a prognostic nomogram based on clinicopathological features and IHC biomarkers in patients with OSCC after surgery.

## Materials and Methods

### Patients and Samples

We retrospectively reviewed the medical records of 301 patients who had undergone surgery with or without induction chemotherapy at Tianjin Medical University Cancer Institute and Hospital between January 2010 and December 2015. A total of 294 patients with complete clinical and follow-up information were enrolled in the study. All patients involved were restaged according to the 8^th^ edition of the AJCC staging system. Using computer-generated numbers, patients were randomly stratified into two groups: a training cohort (147 patients) and validation cohort (147 patients). This study was conducted in accordance with the Declaration of Helsinki. Written-informed consent was obtained from all patients, and the study was approved by the Clinical Research Ethics Committee of Tianjin Medical University Cancer Institute and Hospital.

The inclusion criteria were as follows: (1) patients were primarily diagnosed with OSCC and underwent surgical treatment, (2) patients did not have a history of cancer disease or treatment, (3) patients had complete clinical, pathological and follow-up data, and (4) patients provided written informed consent.

### Procedure of IHC Expression

The tissues were formalin-fixed and paraffin-embedded. To assess the expression of the biomarkers, the IHC assay was performed using the following antibodies: c-Met (25849-1-AP, Proteintech), vimentin (5741, Cell Signaling), E-cadherin (3195, Cell Signaling), N-cadherin (13116, Cell Signaling), MMP9 (ab38898, Abcam), HIF-1α (36169, Cell Signaling), HIF-2α (ab199, Abcam), β-catenin (84803, Cell Signaling), pSTAT3 (9145, Cell Signaling), VHL (ab238681, Abcam), Bcl-2 (ab32124, Abcam), VEGF-a (ab39250, Abcam), VEGF-c (22601-1-AP, Proteintech), EZH2 (5246, Cell Signaling), SUZ12 (ab12073, Abcam), H3K27AC (ab4729, Abcam). Sections (4 μm thick) were cut from formalin-fixed, paraffin-embedded blocks. The paraffin sections were placed in an oven at 65 °C and baked for 1 hour; the paraffin was removed and then the sections were immersed in distilled water following routine methods. To block endogenous peroxidase, the sections were blocked with 3% peroxide-methanol at room temperature. Antigens were retrieved by boiling in citrate buffer (pH 6.0) in a pressure cooker. Serum blocking was performed using 10% normal rabbit serum for 30 min, and then the slides were incubated with the primary antibody overnight at 4 °C. Then, the cells were incubated with a response enhancer and enhanced enzyme labelled goat anti-mouse/rabbit IgG polymer for 30 min at room temperature. Then, 0.05% 3,3′-diaminobenzidine tetrahydrochloride (DAB) was used prior to counterstaining with modified Harris hematoxylin, and hydrochloride alcohol was used for differentiation.

Tumour cell staining was assigned a score according to a previous study 7. For the evaluation of IHC results, all the biomarkers were scored by both the maximal intensity of staining (0, negative; 1, weak; 2, moderate; and 3, strong) and the percentage of positive tumors cells (0%-100%; with any intensity of positive staining) to generate a modified IHC score (IHCs, range: 0-300). All of the biomarkers selected were cytoplasmic and membranous in tumour cells. The IHC results were evaluated by 2 independent practising pathologists who were blinded to the clinical outcome of all cases. A third pathologist was consulted when disagreements occurred between the two pathologists. If the result of the third pathologist agreed with one, then that value was employed. If the opinion of the third pathologist was different, the decision would be cooperatively determined by the three pathologists.

### Calculation of the Cut-off Point and Validation of the Combined IHCs Classifier

The cutoff score for each biomarker was selected based on the association with the patients' survival time by R software (version 3.6.1). The “survival” package of R software version was used. Based on the IHCs of each biomarker, patients were classified into two groups and the association between prognosis and biomarker expression was evaluated. The best expression cut-off referred to the IHCs that yielded the maximal difference with respect to survival between the two groups at the lowest log-rank *P* value. The best cut-off value was selected based on survival analysis.

The least absolute shrinkage and selection operator (LASSO) Cox regression model was used to evaluate high-dimensional data, by shrinking and selecting the biomarkers, that were the most predictive features of patients with OSCC in the training cohort. The LASSO Cox regression model was constructed by R software using the “glmnet” package.

### Statistical Analysis

The two groups were compared using the t-test for continuous variables and chi-square test for categorical variables. We used the Kaplan-Meier survival analysis and log-rank test to estimate the OS. Univariate and multivariate analyses were performed with the Cox regression model. The nomogram was generated using the “rms” package of R software. Receiver operating characteristic (ROC) analysis was conducted to explore the prognostic performance of the IHCs classifier. Decision curve analysis (DCA) was carried out to evaluate the clinical utility of the nomogram. Statistical analysis was performed with R software (3.6.1) and SPSS software (version 25.0). Statistical significance was two-sided, and *P* <0.05 was considered statistically significant.

## Results

### Clinical Characteristics and Selection of Biomarkers

**Figure 2:**
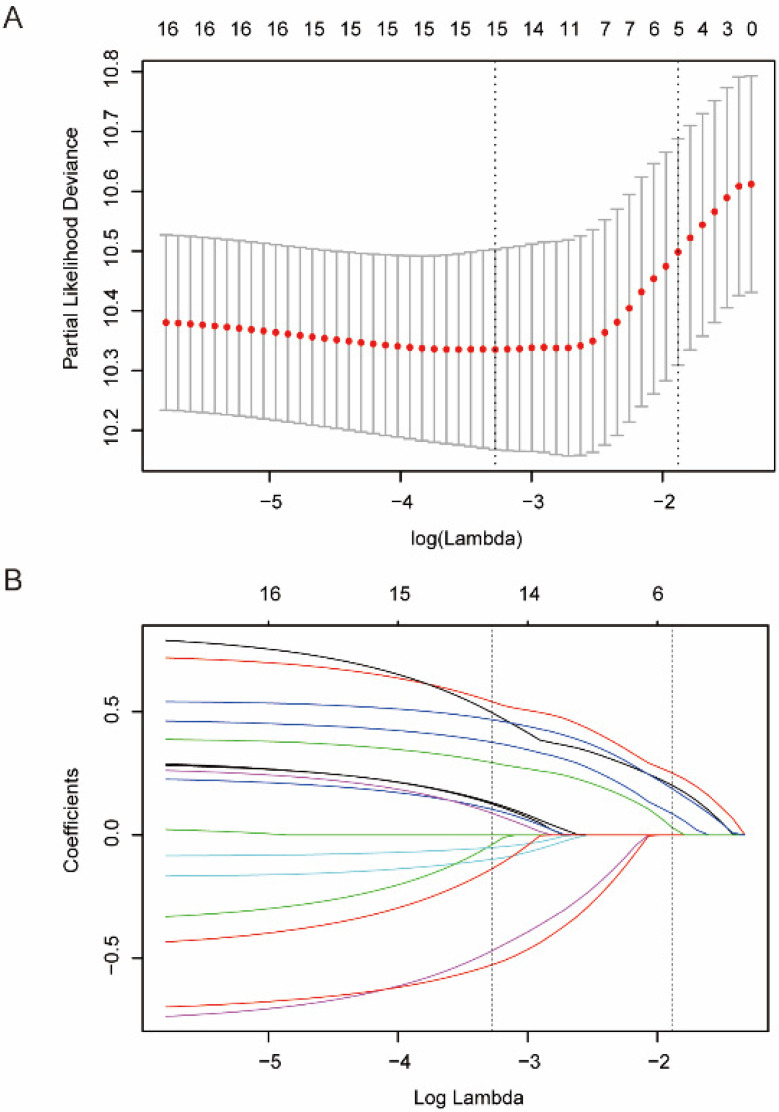
Feature selection using LASSO Cox regression model. **(A)** LASSO coefficient profiles the 16 biomarkers associated with OSCC. **(B)** Tuning parameter selection in the LASSO model. We selected λ via 1-SE (standard error) criteria. A value λ = 0.009 with log (λ) = -4.673 was chosen by 10-fold cross-validation via the 1-SE criteria.

The clinical and pathological characteristics of the patients in the training and validation cohort are listed in Table 1. We used the “survminer” package of R software to determine the cut-off score for the sixteen biomarkers separately (Figure S1). Details are provided in the supplementary materials. We selected five biomarkers of sixteen biomarkers using the LASSO Cox regression model in the training cohort, and these biomarkers were cMet, Vimentin, HIF-2α, VEGF-c, and Bcl-2 (Figure 2). Then, a formula was derived to calculate the score of each patient, based on their individual expression levels of these five biomarkers: IHCs =0.27193962*VEGF-c+0.11069870*HIF-2α +0.22491359*Vimentin+0.06970822*Bcl-2+0.21782503*c-Met. In this formula, low IHC expression equals 0 and high expression equals 1. Then we further divided the 147 patients into a high-IHCs group and a low-IHCs group with an IHCs of 0.383 as the cutoff. In the training cohort, the 5-year OS was 62.1% in low-IHCs group and 28.2% in high-IHCs group (*P*<0.001, Figure 4B). The 5-year OS was 68.6% for the low-IHCs group and 28.4% for the high-IHCs group in the validation cohort (*P*<0.001, Figure 4D). Patients with low IHC expression of have a longer 5-year OS in both the training cohort and validation cohort.

### Prediction Accuracy of the IHCS Group

**Figure 3:**
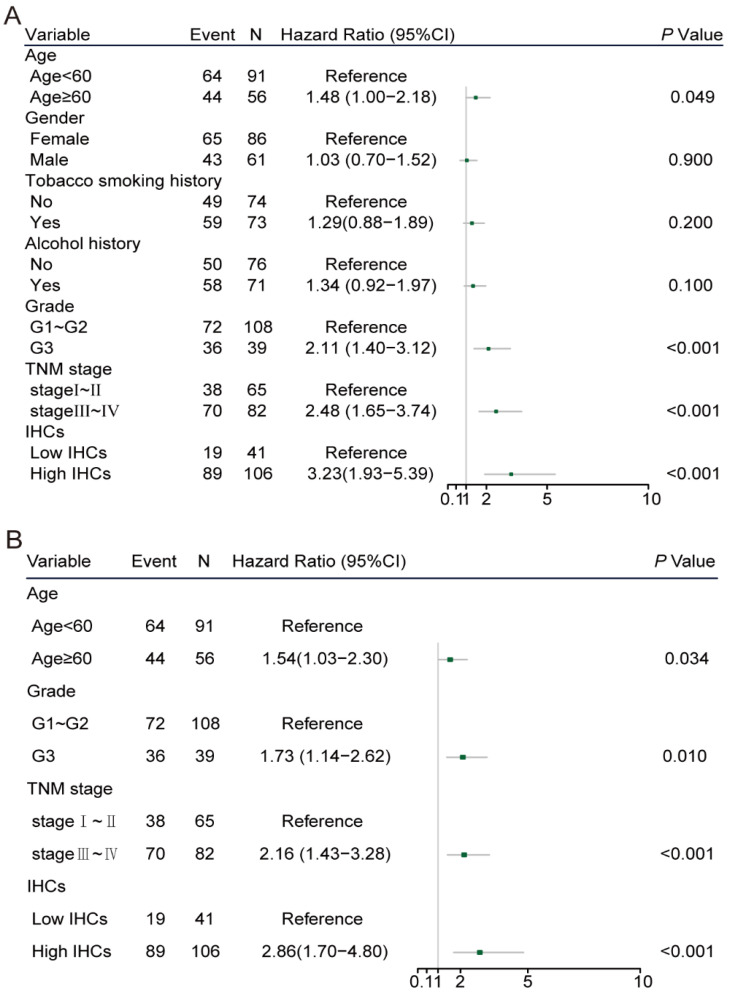

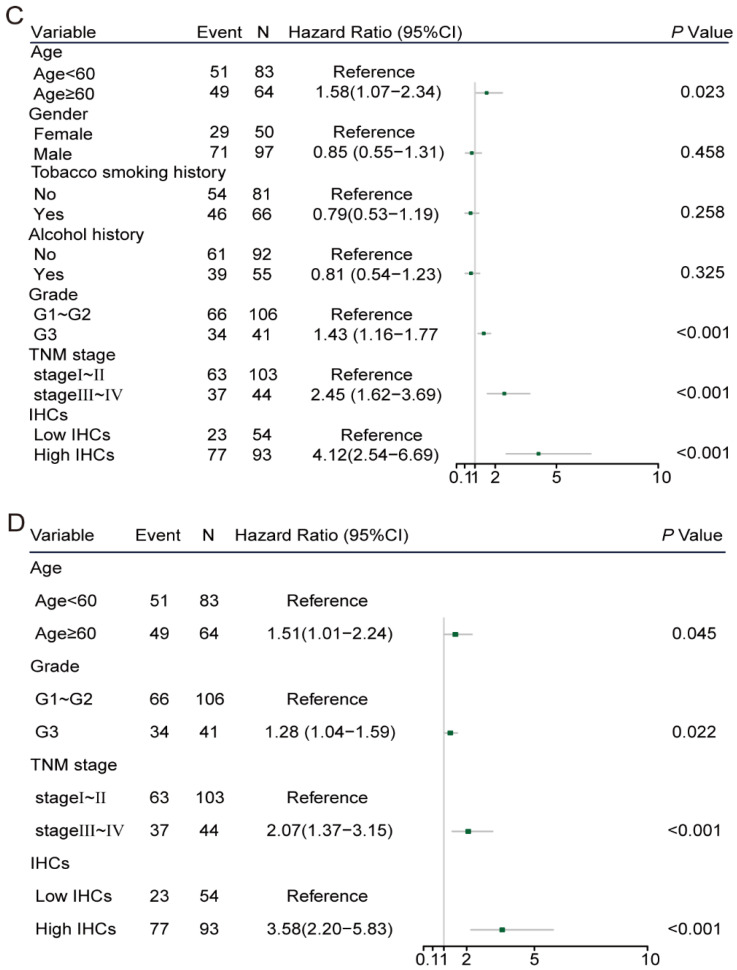
Clinicopathological risk factors for patients with OS.** (C)** Univariate cox regression analysis of patients with OS in the validation cohort.** (D)** Multivariate cox regression analysis of patients with OS in the validation cohort.

**Figure 4:**
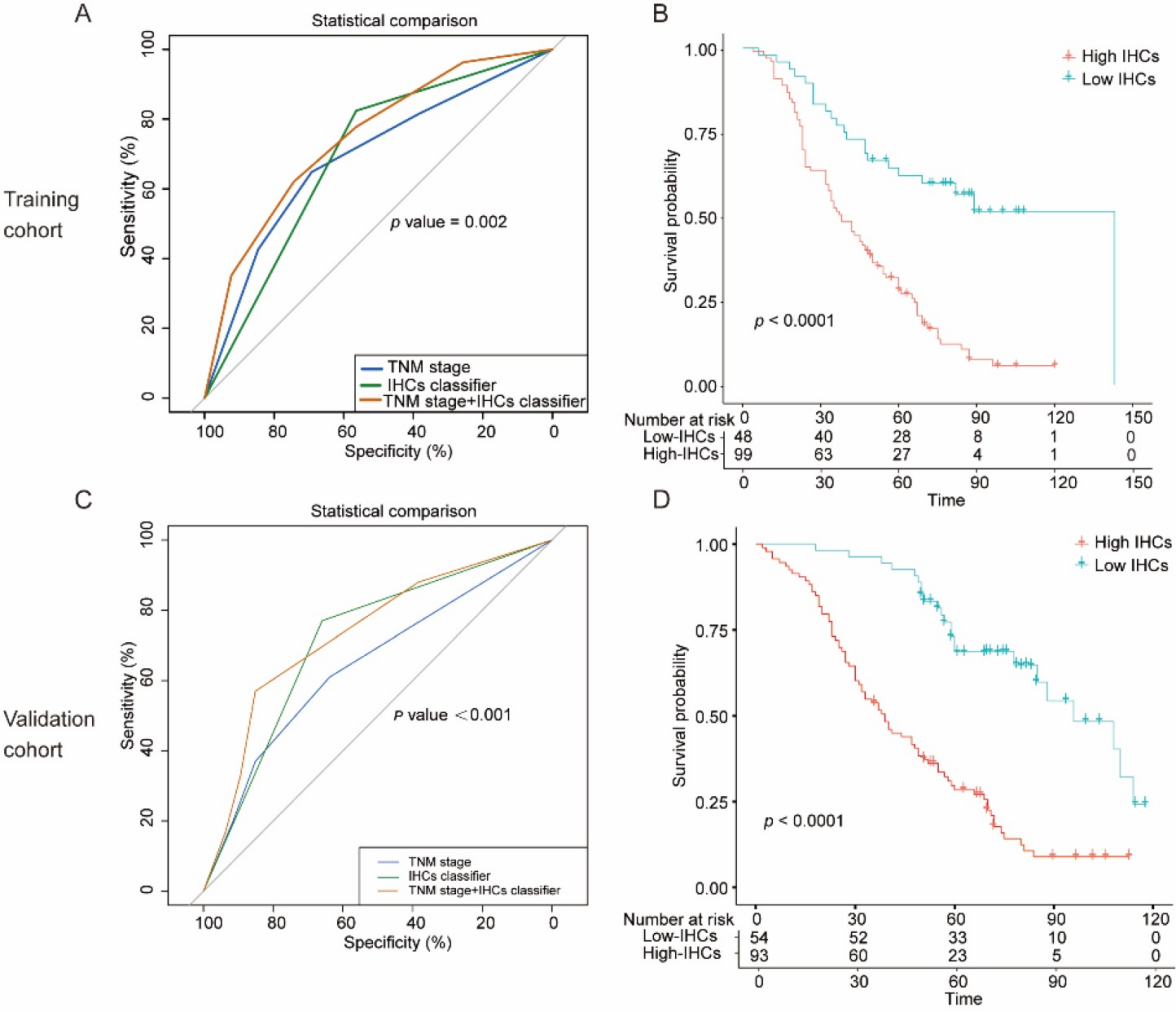
The specificity and significance of the clinical use of the IHCs classier. **(A)** Comparison of the prognostic value of IHCs with TNM staging in the training cohort. **(B)** Kaplan-Meier survival analysis of overall survival according to the IHCs classier in the training cohort. **(C)** Comparison of the prognostic value of IHCs with TNM staging in the validation cohort. **(D)** Kaplan-Meier survival analysis of overall survival according to the IHCs classier in the validation cohort.

In the univariate Cox regression analysis, age≥60 (hazard ratio [HR]: 1.48, 95% CI: 1.00-2.18, *P*=0.049), poor tumuor differentiation (HR: 2.11, 95% CI: 1.40-3.12, *P*<0.001), stage Ⅲ-Ⅳ (HR: 2.48, 95% CI: 1.65-3.74, *P*<0.001) and high IHC classifier expression (HR: 3.23, 95% CI: 1.93-5.39, *P*<0.001) were significantly associated with poor prognosis in the training cohort (Figure 3A). The multivariate Cox analysis identified that the age ≥60 (HR: 1.54, 95% CI: 1.03-2.30, *P*=0.034), poor differentiation (HR: 1.73, 95% CI: 1.14-2.62, *P*=0.010), stage III-IV (HR: 2.16, 95% CI: 1.43-3.26, *P*<0.001) and high IHCs classifier (HR: 2.86, 95% CI: 1.70-4.80, *P*<0.001) were independent prognostic factors for OS in the training cohort (Figure 3B). Similar results were concluded in the validation cohort (Figure 3C, D). The area under the ROC curve (AUROC) of the TNM stage was 0.690 (95% CI: 0.598-0.781), while the AUROC of the combination of the IHCs classifier and TNM stage was 0.746 (95% CI: 0.658-0.833), which showed a better performance for predicting the OS than the TNM stage alone with *P*=0.002 (Figure 4A). Similar to the results displayed in the validation cohort, the AUROC of the TNM stage combined with the IHCs classifier was 0.735 (95% CI: 0.651-0.818), which was higher than that of the TNM stage alone (0.645, 95% CI: 0.559-0.731) (*P*<0.002) (Figure 4C). Therefore, the addition of the IHCs classifier to the TNM group could make it a better tool to predict the survival outcomes for patients with OSCC.

### Construction of the IHCs-based Nomogram

**Figure 5:**
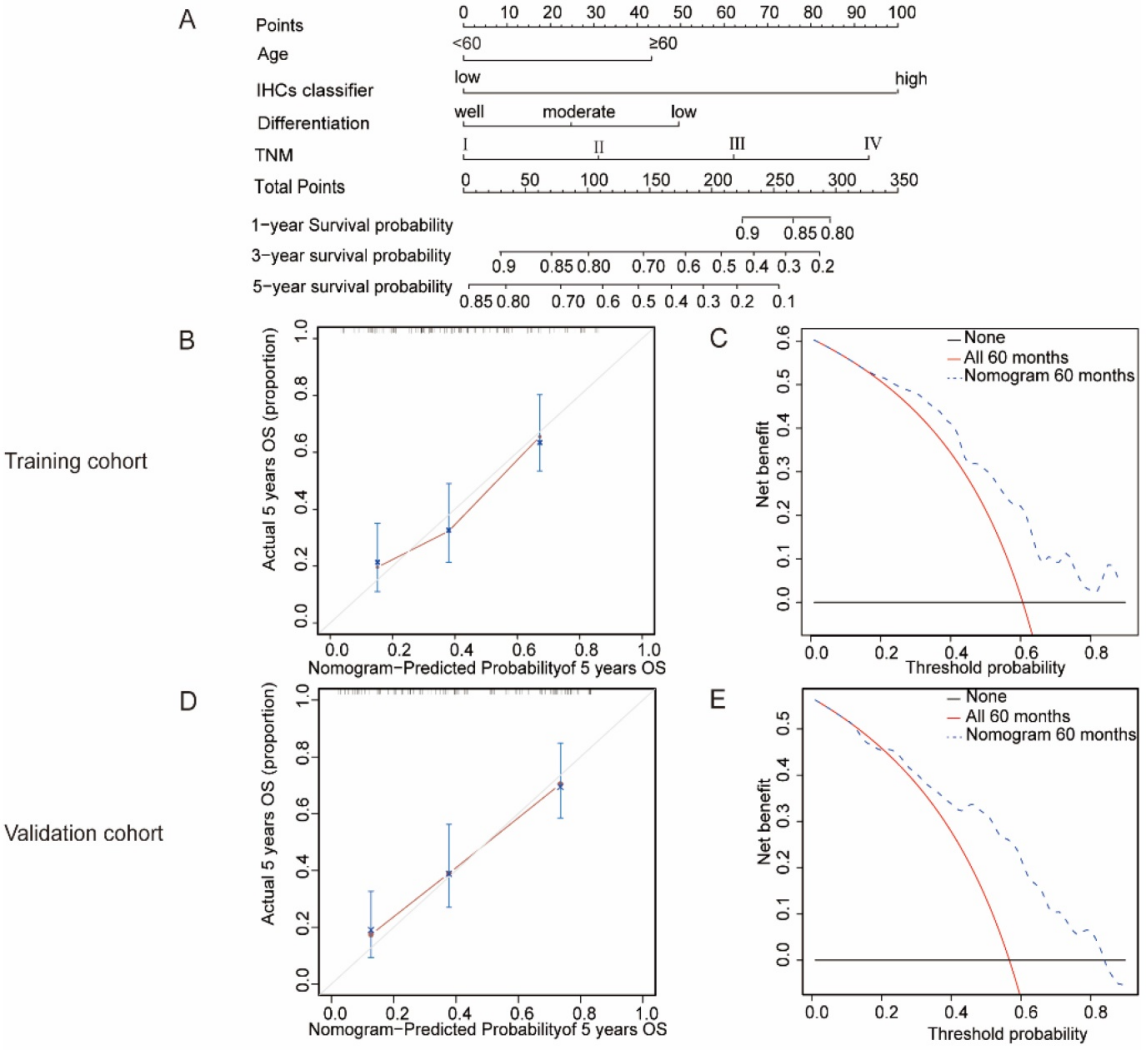
The nomogram to predict 1-year, 3-year and 5-year survival probability for OSCC. (**A**) A nomogram established by the combination of the clinical factors and IHCs. (**B**)(**D**) The calibration curve for predicting patients 5-year OS in the training cohort and validation cohort. **(C)(E)** Evaluation of nomogram using decision curve analysis for the clinical utility of the nomogram in the training cohort and validation cohort.

Therefore, a nomogram was built to integrate the IHCs classifier and clinicopathological factors related to the survival outcomes of the patients with OSCC. The C-index was 0.67 and 0.76 in the training cohort and validation cohort, respectively. The calibration curves showed that our nomogram had a good consistency with the ideal model for predicting the 3- and 5-year OS in the training cohort and validation cohort (Figures 5B, 5D and S2). DCA was presented to assess the value of the clinical unity. The results are presented in Figures 5C, 5E and S3; the x-axis of the graph represented the threshold probability, the y-axis measured the net benefit. This method incorporates the clinical consequences of the nomograms by applying a different weight to the true- and false-positive results. These results showed that our nomogram could help gain more net benefits in clinical practice.

## Discussion

The biological propensity for local invasion and the high rate of cervical lymph node metastasis are usually regarded as the main reasons related to the poor prognosis for patients with OSCC 8, 9. Although the TNM stage system could be used as a tool for predicting the prognosis of patients, it still has some limitations. The different degrees of tumour aggressiveness could be closely related to the molecular subtype, which might result from tumour heterogeneity and indirectly result in different risks of recurrence and metastasis 10. Hence, an accurate evaluation of patients with different survival outcomes could be regarded as an essential progression, thus systemic therapy could also be formulated.

Along with acknowledging tumour heterogeneity, an increasing number of studies have committed to investigating supplements to the TNM staging system for specifically predicting survival outcomes. Nomograms for the prediction of the prognosis in cholangiocarcinoma, colorectal cancer, nasopharyngeal carcinoma and so on have been constructed and published 11-14. Among them, a study generated a distant metastasis gene signature for locoregionally advanced nasopharyngeal carcinoma that consisted of 13 genes to divide patients into high-risk and low-risk groups using microarray analysis, which had an accuracy of 74.8% for predicting distant metastasis-free survival 13. Another study investigated the collagen signature in tumour microenvironment as an indicator of lymph node metastasis in early gastric cancer and validated a nomogram based on the collagen signature and other pathological findings for predicting lymph node metastasis in gastric cancer 15. Meng J et al. 16 constructed a nomogram combining the IHC biomarkers with TNM stage for the predicting recurrence-free survival of patients with oesophageal squamous cell carcinoma. The combination of the markers and TNM stage was proved to be more predictive than the TNM stage alone (AUROC: 0.751, 95%CI: 0.681-0.882 vs AUROC: 0.678, 95%CI: 0.607-0. 750).

Similarly, nomograms have been constructed to predict prognosis for patients with OSCC. Zhou C 17 developed and validated a seven-immune-feature-based prognostic score for patients with OSCC after curative resection. Some nomograms reported in the previous studies assessed the association of preoperative lymphocyte-to-monocyte ratio or some other clinical features with survival in patients with OSCC 18-20. Apart from those studies, there have been some other prognostic nomograms based on mRNA expression profiles from the public databases that improved predicting the prognosis of patients with OSCC 21-23. All of these studies showed promising prospects in clinical applications. However, as far as we know, there have been no reliable molecular profiling features for predicting the prognosis for patients with OSCC. Moreover, some limitations, such as the uncertainty of complicated molecular biology methods, the requirements for fresh tissues and the high cost of examination, exist in gene expression-based methods when applied to the clinical diagnosis. Immunohistochemical staining, as an easy-to-use method, could be applied to conveniently and economically diagnose and determine the expression of tumour-associated proteins. Compared to the previous study, this is the first study to include a relatively large sample size to analyse the IHC expression of biomarkers for patients with OSCC, all of whom received surgical treatment.

Currently, no molecular biomarkers have been clinically used to evaluate the prognosis in patients with OSCC. Researchers have made great efforts on head and neck squamous cell carcinoma before, and found that some biomarkers played an important role in the development of the disease 24-26. Our group have also found that some biomarkers were associated with the prognosis of patients with OSCC 27, 28. However, most of the studies have only investigated one or more biomarkers in a single signaling pathway rather than integrating them together. In this study, we analysed the expression of IHC biomarkers in patients from our medical centre, and built an IHC classifier which could divide patients into high-risk group and low-risk group to help to guide clinical decision making. Even though quite a few patients could get benefit from multimodal treatment involving chemotherapy, radiotherapy and targeted-therapeutics 29, the adverse therapeutic effects still cannot be ignored. The suffering could be alleviated and the chance of survival would be increased if patients received a precise and individualized treatment. Thus, the issue of functional preservation can be considered more when formulating surgical procedures for patients in the low-risk group, so that the quality of life of patients with OSCC could be guaranteed. A more positive treatment, such as accepting a radical surgical approach or receiving more adjuvant therapy, should be implemented when patients are classified into high risk group, consequently the risk of recurrence and mortality could be lowered. Moreover, the financial burden could be reduced for patients with OSCC.

In this study, we investigated sixteen biomarkers that have been reported to be influential on the prognosis of patients with OSCC, and constructed a model named the IHCs classifier, which integrated the expression of the selected five biomarkers via the LASSO Cox regression model. The LASSO Cox regression model could make the IHCs classifier more accurate to predict the prognosis by shrinking the number of biomarkers and eliminating the influence of gene co-expression on the prognosis 30, 31. Although the role and mechanism of the five markers in OSCC was reported in the previous studies, a combination role of the biomarkers could be unclear in the present study. The IHCs classifier with the combination of these five biomarkers could be more precise and scientific for predicting prognosis for patients than that with only one biomarker. Moreover, the small number of biomarkers could make this IHCs classifier convenient to use in our clinical work, and may reduce the economic burden of patients. To better improve the predictive accuracy, a nomogram was built by integrating the clinicopathological characteristics with the IHCs classifier. The AUROC of the combination of the IHCs classifier and TNM stage was 0.746 (95% CI: 0.658-0.833) in the primary cohort and 0.735 (95% CI: 0.651-0.818) in the validation cohort, both higher than the AUROC of TNM stage alone respectively in the primary cohort and validation cohort, indicating that the IHCs classifier could be a good supplement to TNM stage for predicting the prognosis of patients and making individualized treatment decisions.

Cancer is a heterogeneous disease. Exploring dysregulated genes associated with carcinogenesis and development may help improve the prognosis and of patients and treatment strategies. In our study, we included five biomarkers that may work in the nomogram for OSCC. The MET gene encodes the receptor for hepatocyte growth factor (HGF), and the HGF/c-Met signalling pathway, which is overexpressed in various human solid tumours 24, 32-34. A study reported that c-Met positive cells had cancer stem cell properties that could generate tumour cells and make them develop resistance to cisplatin in patients with head and neck squamous cell carcinoma (HNSCC), thereby significantly influencing the prognosis 35. Hypoxia is one of the characteristics of the microenvironment of malignant solid tumours 36. Hypoxia-induced factors (HIFs), which have been demonstrated to exert the effects of an oncogene, have a role in angiogenesis and regulate tumorigenesis of cancer cells 37. Although HIF-2α has been reported to have either a promoting or a suppressing role in different types of tumours 38-40, it has been widely accepted that HIF-2α contributes to the tumour angiogenesis 38, 41. HIF-2α could influence tumour cell proliferation, and apoptosis or tumour angiogenesis by regulating hypoxia-related genes, such as VEGF, cyclin Dl and erythropoietin (EPO) 39, 42. The knockdown of HIF-2α could inhibit VEGF expression 41, 43. In addition, a few studies have shown that HIF-2 may be involved in the tumour resistance to radiotherapy and chemotherapy 44.

Tumour growth and metastasis depend on angiogenesis. VEGF is the most directly related protein influencing angiogenesis. VEGF-C could bind to VEGFR-3, which is mainly expressed in lymphatic endothelial cells, and its affinity for VEGFR-3 is more than three times higher than that for vascular endothelial growth factor receptor-2 (VEGFR-2). Therefore, VEGF-C could be a major inducer of lymphangiogenesis in various malignancies including OSCC 45, 46. In addition, VEGF-C has been reported to be correlated with lymphatic vessel density and microvessel density 47, and it is an indicator for lymph node metastasis, and tumour recurrence 48. The Bcl-2 gene is one of the most important genes regulating apoptosis by suppressing apoptosis and prolonging cell survival 49. Our previous study showed that knocking down Bcl-2 might initiate cascade response and change the EMT process and suppress cell migration and invasion in OSCC 27. Similarly, Arshad Rahman et al. 50 indicated that the expression of Bcl-2 was higher in patients with stage III/IV or poorly differentiated tumour. It has been demonstrated that EMT plays a critical role in tumour invasion and metastasis 51. Vimentin, a mesenchymal-specific protein, is generally expressed during the process of EMT, but is not induced in normal epithelial cells. It has also been found that vimentin was one of the most upregulated genes in metastatic OSCC cells, and that high expression of vimentin was correlated with lymph node metastasis and poor prognosis in patients with OSCC 52.

To the best of our knowledge, this is the first study to include 5 biomarkers which were selected from 16 biomarkers that have been reported to be involved in biological processes during carcinogenesis, and construct a nomogram involving a combined multi-biomarker classifier for patients with OSCC. However, our study has some limitations. Our research is a retrospective study in a single cancer centre in North of China. The information being involved in the studies could not be representative for patients from all over the world. This study could be more persuasive if an external validation cohort was included. Another limitation is that the 16 biomarkers involved are based on the previous research of our team and the current recognition of the mechanism of OSCC. As OSCC research develops, more specific and relevant biomarkers need to be investigated. Thus, prospective research including more biomarkers and patients from multiple centres will be needed to validate our findings.

In conclusion, the nomogram showed good predictive accuracy and clinical unity for predicting the prognosis of patients with OSCC, and it could be a potential tool for guiding the individual decision-making process.

## Supplementary Material

Supplementary figures.

## Figures and Tables

**Figure 1:**
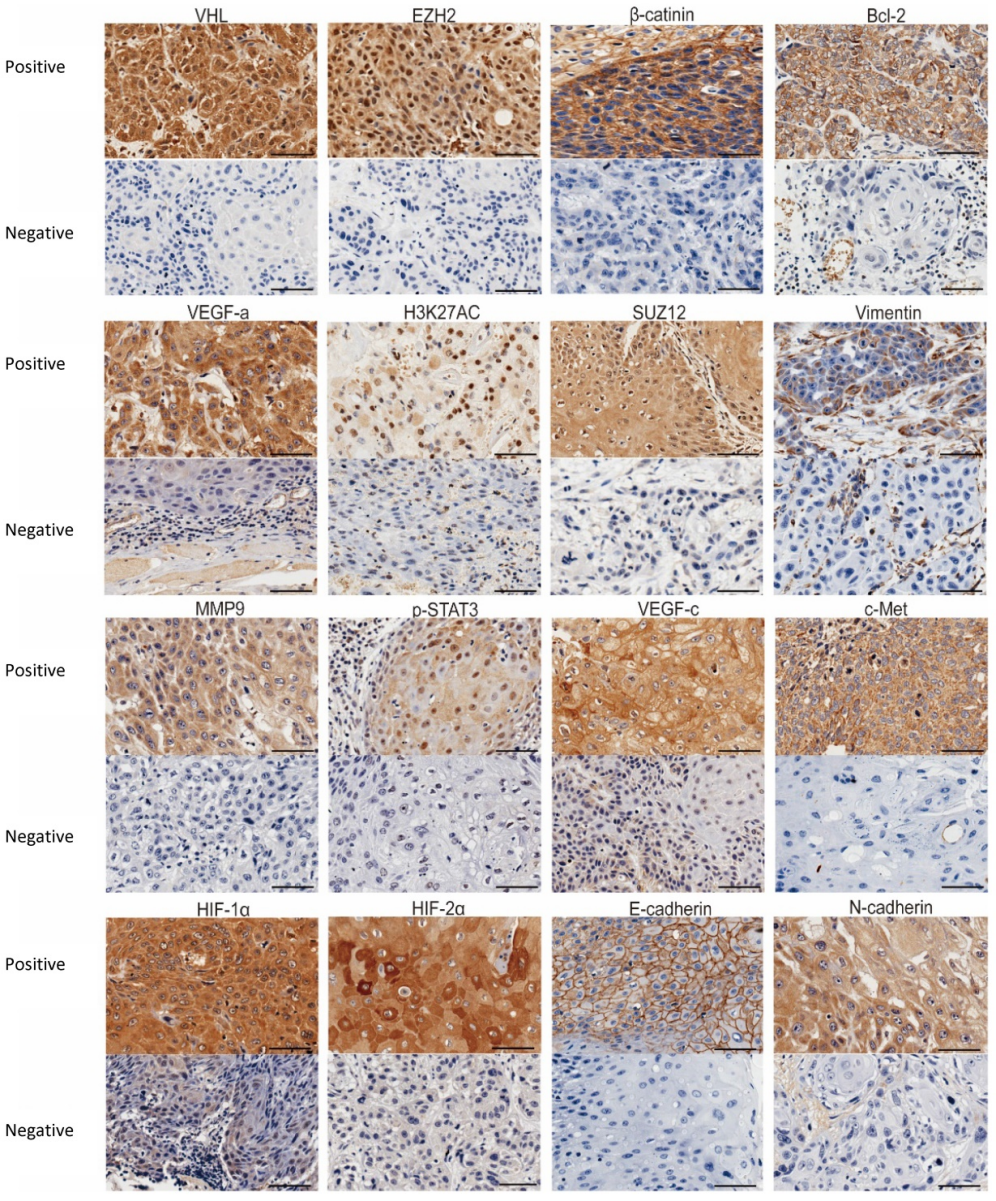
Representative IHC images of the 16 biomarkers expression. Bar, 100 um.

**Table 1:** Clinical and pathological characteristics of patients according to the IHCs in the training and validation cohorts

Variables	Training Cohort (n=147)				Validation Cohort (n=147)			
	N	Low-IHCs	High-IHCs	*P*	N	Low-IHCs	High-IHCs	*P*
**Sex**								
Male	86	21 (51.2)	65 (61.3)	0.265	97	37 (68.5)	60 (64.5)	0.621
**Female**	61	20 (48.8)	41 (51.2)		50	17 (31.5)	33 (35.5)	
Age								
<60	91	25 (61.0)	66 (62.3)	0.885	83	34 (63.0)	49 (52.7)	0.226
≥60	56	16 (39.0)	40 (37.7)		64	20 (37.0)	44 (47.3)	
**Depth of invasion**								
T1+T2	92	30 (73.2)	62 (58.5)	0.099	128	51 (94.4)	77 (82.8)	0.042
T3+T4	55	11 (26.8)	44 (41.5)		19	3 (5.6)	16 (17.2)	
**Lymph Node Metastasis**								
N0	90	31 (75.6)	59 (55.7)	0.026	117	47 (87.0)	70 (75.3)	0.088
N1+ N2+ N3	57	10 (24.4)	47 (44.3)		30	7 (13.0)	23 (24.7)	
**TNM Stage**								
I+II	65	25 (61.0)	40 (37.7)	0.011	103	44 (81.5)	59 (63.4)	0.021
III+IV	82	16 (39.0)	66 (62.3)		44	10 (18.5)	34 (36.6)	
**Grade**								
G1-G2	108	31 (75.6)	77 (72.6)	0.715	106	46 (85.2)	60 (64.5)	0.007
G3	39	10 (24.4)	29 (27.4)		41	8 (14.8)	33 (35.5)	
**Smoke**								
Yes	73	21 (51.2)	52 (49.1)	0.814	66	31 (57.4)	35 (37.6)	0.023
No	74	20 (48.8)	54 (50.9)		81	23 (42.6)	58 (62.4)	
**Drink**								
Yes	71	20 (48.8)	51 (48.1)	0.942	55	23 (42.6)	32 (34.4)	0.347
No	76	21 (51.2)	55 (51.9)		92	31 (57.4)	61 (65.6)	

## Abbreviations

OSCC: oral squamous cell carcinoma
OS: overall survival
AJCC: American Joint Committee on Cancer
DOI: depth of invasion
IHC: Immunohistochemistry
EMT: epithelial-mesenchymal transition
DAB: 3,3′-diaminobenzidine tetrahydrochloride
IHCs: IHC score
LASSO: least absolute shrinkage and selection operator
ROC: Receiver operating characteristic
DCA: Decision curve analysis
HR: hazard ratio
AUROC: area under the ROC curve
HGF: hepatocyte growth factor
HNSCC: head and neck squamous cell carcinoma
HIFs: hypoxia-induced factors
EPO: erythropoietin
VEGFR-2: vascular endothelial growth factor receptor-2
