# Supplementary material for: Development and Validation of a Nomogram based on cell growth-related Biomarkers for Oral Squamous Cell Carcinoma
Source: J Cancer. 2021 Jun 22;12(17):5153–63. doi: 10.7150/jca.54475 (PMC8317514; doi:10.7150/jca.54475)
Supplement: Supplementary file 1 — Supplementary figures. [file jcav12p5153s1.pdf]

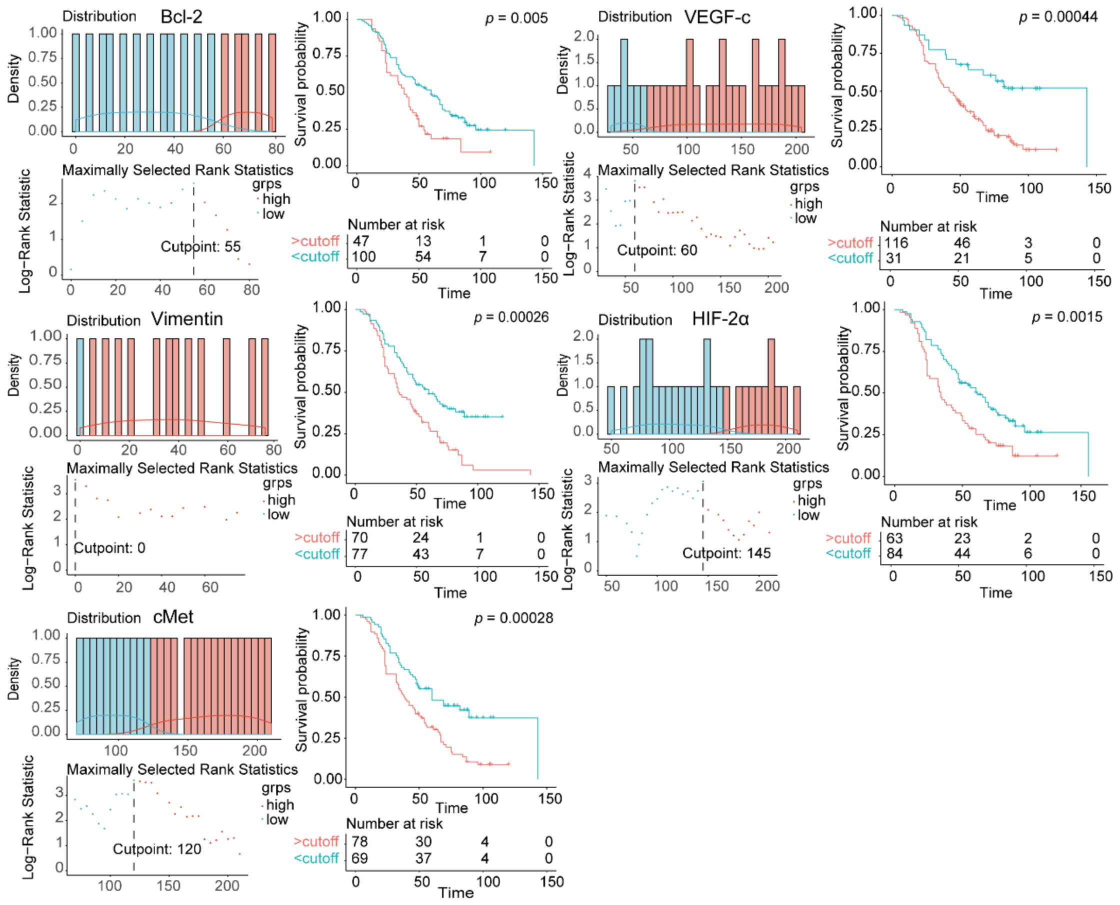

Figure S1. The selected cut off value analyzed by R software and the Kaplan-Meier survival analysis of the 16 biomarkers.

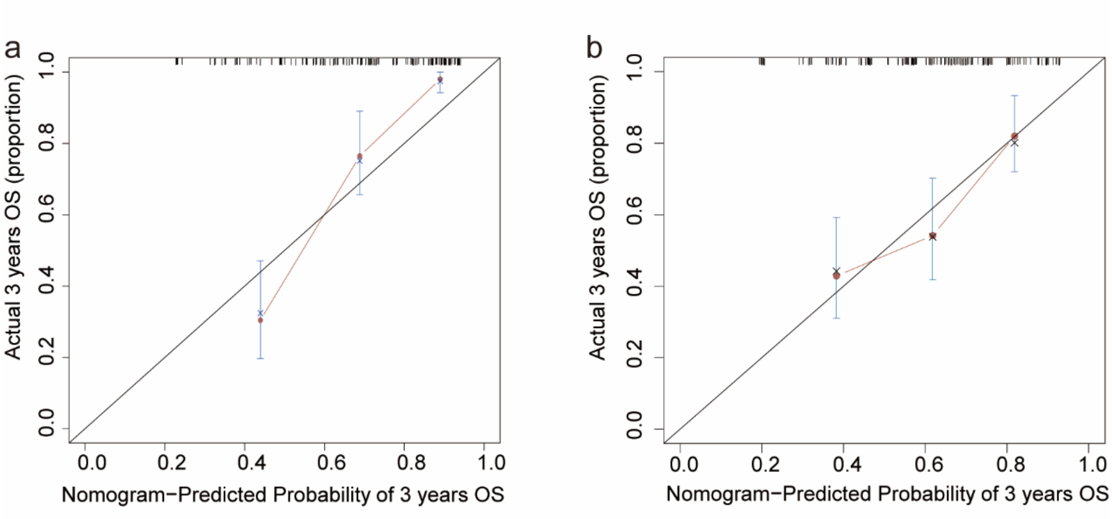

Figure S2. Decision curve analysis of the 3-year OS nomogram in patients with OSCC cancer in the training cohort and validation cohort. The net benefits (y axis) as calculated in the training cohort **(A)** the validation cohort **(B)** are plotted against the threshold probabilities of 3-year OS on the x axis. The dashed line represents the nomogram; The solid line: to assume all patients to have dead; horizontal black line: to assume no patients to have dead.

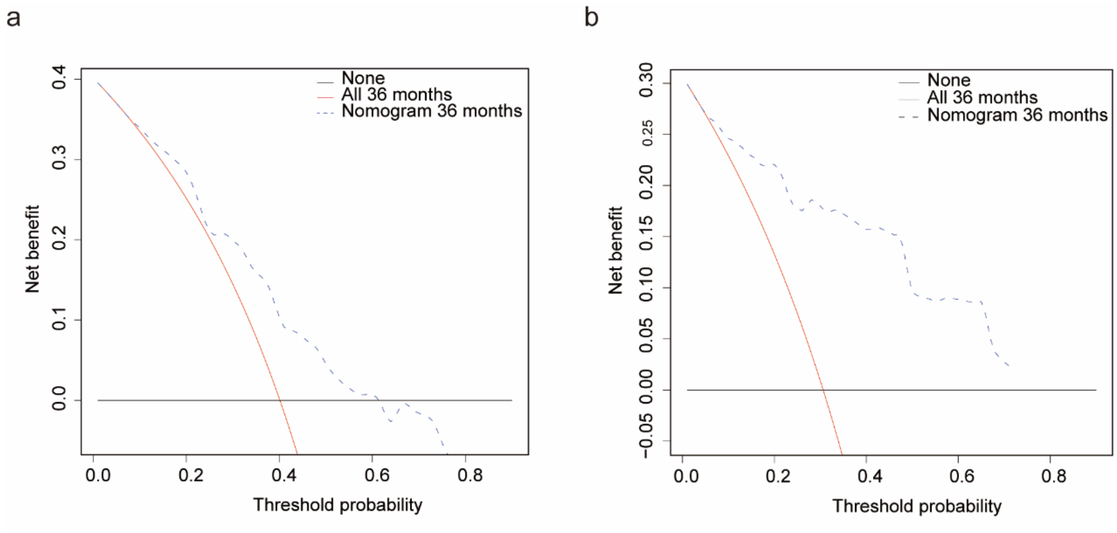

Figure S3. Evaluation of the 3-year OS nomogram using calibration curves. The dashed line represents an ideal evaluation, whereas the red line represents the performance of the nomogram in the training cohort **(A)** and validation cohort **(B)**.
